# Influence of Motor and Cognitive Tasks on Time Estimation

**DOI:** 10.3390/brainsci12030404

**Published:** 2022-03-18

**Authors:** Serena Castellotti, Ottavia D’Agostino, Alessandra Biondi, Luigi Pignatiello, Maria Michela Del Viva

**Affiliations:** 1 Department of Neurofarba, University of Florence, 1035 Florence, Italy; serena.castellotti@unifi.it (S.C.); ottavia.dagostino@stud.unifi.it (O.D.); alessandra.biondi@stud.unifi.it (A.B.); 2 Department of Medicine and Surgery, University of Parma, 43126 Parma, Italy; luigi.pignatiello@studenti.unipr.it

**Keywords:** time perception, duration estimation, cognitive load, walking, clock speed, cognitive–motor interaction

## Abstract

The passing of time can be precisely measured by using clocks, whereas humans’ estimation of temporal durations is influenced by many physical, cognitive and contextual factors, which distort our internal clock. Although it has been shown that temporal estimation accuracy is impaired by non-temporal tasks performed at the same time, no studies have investigated how concurrent cognitive and motor tasks interfere with time estimation. Moreover, most experiments only tested time intervals of a few seconds. In the present study, participants were asked to perform cognitive tasks of different difficulties (look, read, solve simple and hard mathematical operations) and estimate durations of up to two minutes, while walking or sitting. The results show that if observers pay attention only to time without performing any other mental task, they tend to overestimate the durations. Meanwhile, the more difficult the concurrent task, the more they tend to underestimate the time. These distortions are even more pronounced when observers are walking. Estimation biases and uncertainties change differently with durations depending on the task, consistent with a fixed relative uncertainty. Our findings show that cognitive and motor systems interact non-linearly and interfere with time perception processes, suggesting that they all compete for the same resources.

## 1. Introduction

Time can be defined as a continuous sequence of events that occur from the past through the present to the future. It is not something we can see, touch or taste, but we can measure its passage in seconds, minutes, hours, days and so on, thanks to clocks and calendars.

Time perception refers to a person’s subjective experience of the passage of time [1]. The way in which we code temporal information has been under debate for a long time and is still little understood.

The most widespread theoretical model of time perception is the *scalar timing theory* [2,3,4,5], which claims that temporal judgments are based on three different stages. Firstly, there is a pacemaker that produces pulses at a regular rate, then a switch that controls the transmission of the pulses to the final stage, and finally, an accumulator that counts them. The content of the accumulator (i.e., the elapsed time) is finally transferred and stored in the working memory (WM) system. The temporal estimation consists of a comparison between the current content of working memory and the contents stored in the long-term memory [6,7,8]. In this framework, attention mechanisms play a fundamental role. Indeed, attention is considered to be the moderator of the on/off switch in internal clock models ([9,10,11]; for reviews, see Refs. [5,12,13,14]). Consistently with Weber’s law, the variability (standard deviation) in estimates (i.e., the sensitivity to time) increases linearly with the length of intervals to be timed (scalar property) (for reviews, see Refs. [3,13,14]).

Beside time theories, experimental studies measuring time estimation make it clear that the perceived duration of events differs significantly from person to person and that each person’s time perception is affected by multiple internal and contextual factors.

Widely studied physical factors that influence subjective time perception are body temperature and physiological arousal [15,16,17]. For example, studies have reported that increasing body temperature leads to an underestimation of time [18,19]. Additionally, increasing arousal lengthens the perceived duration of events, whereas its decrease shortens duration estimation [20,21]. Other studies have demonstrated that influencing factors are stress and anxiety [22,23,24], sleep [25,26], drugs intake [27] and biological variables, such as age [28] and gender [29] (for a review, see Ref. [30]).

Furthermore, some level of experience in particular fields, which involved time counting, such as musical or sport expertise, could influence time estimation accuracy across different groups of individuals (for a review, see Ref. [30]). For example, musicians show very low biases for duration reproduction compared to individuals with no musical training [31,32].

A critical contextual calibration is the central tendency effect, which leads to overestimating short intervals and to underestimating long ones [33,34]. Central tendency effects can be explained by the Bayesian computations in the estimation of magnitudes, for which duration judgments are derived not only from current sensory inputs but are also influenced by the acquired statistics of the distribution of previously experienced durations [34,35,36]. Contextual factors affecting time perception also include familiarity and numerosity of perceived stimuli [37]. For example, the less familiar the stimuli occurring within an interval, the longer they are perceived to be, because they require more storage space. Additionally, perceived durations lengthen as the number of elements contained in the intervals increases [38].

Temporal estimation can be affected by non-temporal tasks performed at the same time, which impairs estimation accuracy. Indeed, several studies have demonstrated that the probability of judging stimulus duration to be short or long is related to the amount of attention devoted to time estimation task. In fact, as theorized by the *attentional allocation model*, paying attention only to time induces temporal overestimation, whereas paying attention away from time in favor of other cognitive tasks causes an underestimation of time ([9,39,40,41,42]; for reviews, see Refs. [13,37,43]). For example, Brown (1997) found that different types of tasks (such as visual search, pursuit rotor tracking and mental arithmetic tasks) induce duration underestimation, which increases as the difficulty of the task increases. On the other hand, temporal production tasks only impair the performance of mental arithmetic [44]. This result can be explained by the working memory model, according to which two concurrent tasks, both requiring the central executive, compete for the same resources, causing mutual interference [44,45,46,47,48].

A recent study by Polti and colleagues (2018) attempts to discern the role of WM and attention in time perception [49]. Participants had to estimate time intervals in the range from 30 s to 90 s, while they fully attended to time (single task), or while performing concurrent cognitive tasks of increasing difficulty (dual task). In the single-task condition, time durations were overestimated compared to the dual-task condition. In the latter, a significant underestimation, proportional to WM load, was found across all the three durations. Moreover, in the dual-task condition, underestimation scaled with duration (i.e., the longer durations were more underestimated than the shorter ones) [49].

There is a consistent body of evidence showing that cognitive and motor tasks mutually interfere (for a review, see Ref. [50]). For example, maintaining standing balance control while concurrently performing math subtractions has a damaging effect on cognitive performance and decreases speed and accuracy of responses [51]. Furthermore, it has been found that counting backward aloud during walking leads to an impairment in the gait maintenance task [52].

Some studies also suggest that time perception and the motor system are related [53,54,55]. Particularly, the way in which time estimation can be modulated by the presence of fine arm movements has been investigated, finding that prolonged movements cause an expansion of the perceived duration of the concurrent stimulus [56]. However, the effects of gross body movements on time estimation have not been systematically studied yet. The only studies, using temporal reproduction paradigms and short time intervals (less than 10 s), have found an overestimation of temporal durations while walking, speculating that movement speeds up the internal clock [57,58].

A large body of studies conducted in the field of human time perception are restricted to estimations of brief time intervals, order of 10–100 ms or order of seconds, while a few explored temporal estimations of intervals in the range of minutes (for reviews, see Refs. [3,13,14,59,60]). Particularly, some studies tested the effects of cognitive and attentional tasks on time estimation of ecologically valid time scales (order of 10–100 s) ([49,61]; for reviews, see Refs. [12,13,37,43]).

Time estimation has also been shown to depend on the specific paradigm at hand. Indeed, it has been shown that in a prospective paradigm, in which participants are aware that they must estimate time, cognitive effort decreases the subjective-to-objective duration ratio. Instead, in a retrospective paradigm, in which participants are not aware of judgment duration task, cognitive load increases the subjective time perception. These opposite effects could be explained by a difference in the processes (especially attention and memory) taking place in prospective and retrospective duration judgments [37].

In the present study, for the first time, we measure prospective estimation of long time intervals (from 13 s to 132 s) while observers walk, to broaden time perception knowledge and fill some of the gaps in the literature. Participants performed four different cognitive tasks of increasing difficulty (look, read, solve simple or hard mathematical operations) in two different motor conditions (walking on a treadmill or sitting on a chair). They were then asked to verbally report how much time had passed from the start to the end of the task. Although the best method to test interval timing in humans is still debatable [3,62,63], verbal estimation is the most ecological way to test the passing of time. Additionally, we purposedly chose a verbal estimation task, instead of more common production/reproduction motor tasks, to avoid possible interferences with the motor condition.

By combining cognitive and motor tasks, we can gain insight on how these different systems interact and influence time perception.

## 2. Materials and Methods

### 2.1. Participants

Sixteen young adults (eight males and eight females; mean age = 25.4 ± 0.7 years) participated in the experiment. All participants were naive as to the purpose of the study and had given written informed consent prior to participation. They were also required to possess a valid medical certificate for the physical activity involved. Before the experiment, all participants had to fill out a questionnaire regarding personal data, expertise in some specific fields (e.g., sport or music), sleeping habits, sleep–wake cycle, presence of optical defects, history of brain damage, heart or respiratory disease, motor diseases, neurological problems, medication intake (e.g., psychotropic drugs or sleeping pills), psychological disorders (e.g., anxiety, depression), physical injuries, dyscalculia or any relevant pathologies. All selected participants had a normal or corrected-to-normal vision, no history of physiological or specific learning disorders and were not on any type of medication. All of them reported to have regular sleep–wake cycle and 7.5 h average night sleep duration (SE = 0.15). None of our participants is a professional athlete or musician. Additionally, before starting each session, we asked participants to rate their mental and physical tiredness with a 7-point Likert scale, obtaining an average value of 1.6 (SE = 0.06). Finally, participants were required to rate their math ability and math anxiety with a 7-point Likert scale. All of them reported having good ability in performing mathematical operations (mean score = 5.2, SE = 0.3), and none of them reported high math anxiety (mean score = 2.2, SE = 0.3). Participants were suggested to wear light sporting clothes and comfortable shoes during the walking sessions and to show up well rested (average night sleep = 7.3 ± 0.07 h before the experimental sessions), not having used exciting substances (e.g., alcohol) the night before the experiment.

The study was conducted according to the guidelines of the Declaration of Helsinki and approved by the local ethics committee (“Commissione per l’Etica della Ricerca”, University of Florence, 7 July 2020, n. 111).

### 2.2. Apparatus and Set-Up

**Figure 1:**
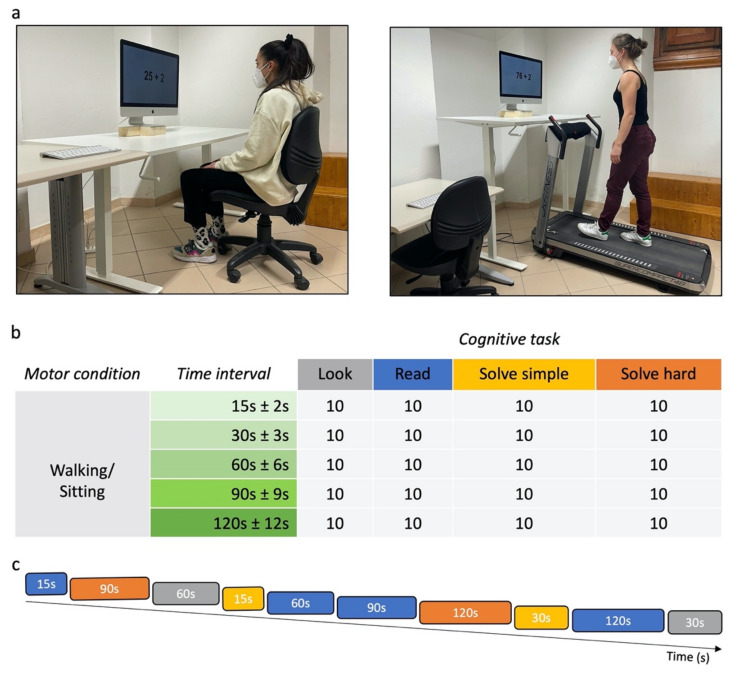
Experimental design. (**a**) Experimental set-up in sitting (**left panel**) and walking condition (**right panel**). (**b**) Number of trials per condition, time interval and cognitive task. Observers performed 10 trials for all possible combinations of time intervals and cognitive tasks in each motor condition. (**c**) Trial randomization in a block. Example of a block of 10 trials. Grey: *look* task; blue: *read* task; yellow: *solve simple* task; orange: *solve hard* task.

Stimuli were programed and displayed on an iMac Retina 5K 27-inch (mid 2015, 3.3 GHz Intel Core i5 processor, MacOs Mojave software 10.12.6 (Cupertino, California), frame rate 60 Hz, 5120 × 2880 pixel resolution). In the sitting condition, observers sat in front of the display (60 × 33.5 cm), subtending 60° × 33.5° of visual angle at a viewing distance of 57 cm (Figure 1a—left panel).

In the walking condition, observers were positioned on a JK Fitness treadmill (Supercompact 48 model, 48 × 130 cm walking belt), 80 cm away from the display, subtending 43° × 24° of visual angle (Figure 1a—right panel). Given the different viewing distances in the two motor conditions, all stimuli sizes were adjusted to subtend the same degrees of visual angle in both conditions.

In both conditions, the experimental procedure was carried out in a temperature-controlled room (25° during the whole experiment). Participants’ responses were entered on a computer keyboard by the experimenter. The experimenter measured the participants’ head temperature through a non-contact infrared thermometer (Berrcom, JXB-178 model). Stimulus presentation and data collection programs were developed using the Psychophysics Toolbox extensions [64,65,66] for Matlab (R2020b version; Natick, Massachusetts: The MathWorks Inc.).

### 2.3. Procedure and Tasks

The whole experiment required four morning sessions (two for each motor condition) of about 2 h each, on different days. Conditions order was randomized across observers (7 participants performed the walking sessions first). Each participant took part in 200 trials for both motor conditions.

For each condition, time estimation of five different time intervals during four different tasks (look, read, solve simple, solve hard) was tested in all participants (Figure 1).

Time intervals were centered on 15, 30, 60, 90 and 120 s to allow comparisons of average estimates with previous studies that used this gross duration discretization [49]. However, the actual duration of each trial varied randomly in a 10% range of its central value but always taking an integer value of seconds. For example, the 15 s nominal time interval could take actual values of 13, 14, 15 16, 17 s. This allows less predictability in time estimation and favors continuous and non-discretized observer responses.

Each possible combination of time interval and task was tested ten times per condition (Figure 1b). The trials were divided in blocks of ten, each containing every time interval twice, so that blocks had about the same duration (~12 min). Such short duration prevents participants from becoming tired. The tasks were randomly distributed across such blocks (see, for example, Figure 1c).

In the walking condition, the treadmill speed was not constant to prevent the participants from counting based on pace. In each block, a preset sequence of speeds, ranging from 1.4 km/h to 3 km/h, with changes ranging from 0.4 to 1.6 km/h every 20–40 s, was used for all observers. Before starting the experiment, observers were allowed to familiarize themselves with speeds and their changes used in the experiment.

To avoid physical and/or mental stress, in both conditions participants were allowed to take short breaks (of about 5 min) between blocks and a longer break (20 min maximum) to rest in the middle of the session (between the fifth and the sixth block). At the beginning and the end of each block, the experimenter measured participants’ body temperature and asked them to rate their physical fatigue and mental tiredness on a 7-point Likert scale. The experimenter also checked the room temperature every 30 min and ensured that it remained stable throughout the whole session.

**Figure 2:**
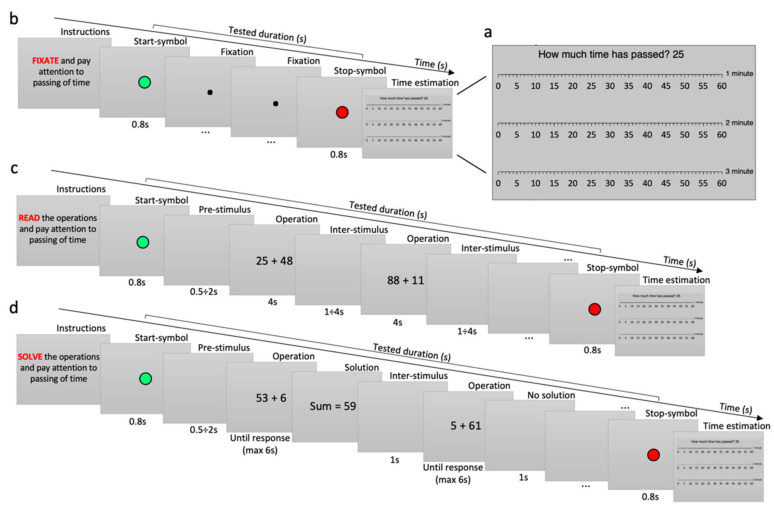
Procedure and tasks. (**a**) Time ruler. Ruler shown at the end of each trial to allow participants to express how much time had passed while they were performing the task. In the example shown, the participant estimated 25 s. (**b**) *Look* task. (**c**) *Read* task. (**d**) *Solve* task. Example of *solve simple* task. The *solve hard* task follows the same procedure but consists of harder mathematical sums. Red words report the task to be performed in the trial. The green circle informs the observers to start estimating the passing of time; the red circle informs the observers to stop estimating the passing of time.

At the beginning of each trial, written instructions about the task to be performed were presented on a gray background (Figure 2). As soon as the participant reported being ready to start, the trial started with the appearance of a green circle (2 × 2°) in the center of the screen (800 ms), which told the observer to start estimating the passing of time. Then, the task began (specifics of each task are described in the paragraph below). At the end of the trial (whose duration was obviously unknow to the observer), a red circle (2 × 2°) appeared in the center (800 ms), informing the observers to stop temporal estimation. Then, a “time ruler” appeared on the screen, showing three scales of seconds from 0 to 60, one for each minute, along with the question “How much time has passed?” (Figure 2a). Observers were required to estimate how much time had passed between the start and stop signals, and their response appeared under the question, as the experimenter typed it in the computer. This graphic representation of time was used to make observers more inclined to give precise and diversified responses in terms of seconds, avoiding gross discretization of answers (such as “10 s”, “½ min”, “1 min”, etc.).

Between the start and stop signal, observers were instructed to perform one of the following tasks requiring different amounts of cognitive load:

“*Look*” task: participants were instructed to look at a fixation point (0.2 × 0.2°) and pay attention to the passing of time. This task did not require any cognitive effort of the observers, who could potentially count the seconds (Figure 2b).

“*Read*” task: participants were required to read aloud some math operations written on the screen and pay attention to the passing of time. Stimuli were black-arial font numbers (5 × 3°) presented on a gray background for 4 s. The first operation was presented after a random period of time from the start signal (500 ms–2 s), and all were alternated with a blank screen of random duration (from 1 to 4 s), to avoid time counting based on the number of stimuli presented. This task was introduced as “attention control”, since reading activity might interfere with time counting, while requiring a minimum cognitive load and sustaining observers’ attention to the cognitive task (Figure 2c).

“*Solve simple*” task: participants were required to solve simple sums of a 1-digit number plus a 2-digit number (without carry over, e.g., 24 + 5) and pay attention to the passing of time. The result of the operations was never higher than 100. The first operation was presented after a random period from the start signal (500 ms–2 s). Operations (5 × 3°) were presented on a gray background until the observer reported aloud the solution or for a maximum of 6 s if no solution was provided. As soon as the observer reported the solution, the experimenter pressed a key to record the response time. The solution was then displayed on the screen while the experimenter typed it in the computer. Then, a blank screen was presented for 1 s before the next operation. This task requires a modest amount of processing load and cognitive effort, which make counting time explicitly very unlikely (Figure 2d).

“*Solve hard*” task: participants were required to solve hard sums of 2-digit numbers (e.g., 37 + 48) and pay attention to the passing of time. Numbers to be summed were never greater than 50. The procedure was the same as for the *solve simple* task. This task requires concentration and high cognitive effort, which prevents time counting.

### 2.4. Data Processing and Statistical Analysis

For each trial, we recorded both the true time and the time estimated by the participant. From these values, the time difference in seconds between effective and estimated durations (estimation bias) was determined. For solve tasks, we also measured the percentage of correct responses and the average response time.

To check the normality of data distributions, Shapiro–Wilks tests were performed for each task and time interval. Since most of those tests revealed deviations from normality, non-parametric statistical tests were used where needed, and an outlier-removal procedure was applied when estimating variances from data to get rid of the longer tails.

To filter the outliers, we first determined a pseudomedian estimate of each distribution by evaluating the Hodges–Lehman statistics; then, we dropped all data deviating by more than 3 SD above or below this pseudomedian [67]. All averages and variances used in our analysis were then calculated on this filtered sample. When a variance of our variance estimate was needed, we estimated it by a bootstrap procedure of the whole process (including the filtering stage), in order to achieve the best accuracy and robustness in spite of the presence of the non-gaussian tails.

To measure with the least squares fitting methods how estimation biases or their uncertainties change with duration, data were averaged over smaller time intervals (2–3 sec) to avoid artificially inflating the variance of data with the effect of the drift of the average estimate within the five larger bins mentioned above.

Below, we reported a detailed list of tests performed to measure different effects.

To assess the effect of all factors playing a role in our study, we performed a linear mixed-effects model with estimation as outcome variable, and cognitive task (look, read, solve simple, solve hard), time interval (15 s, 30 s, 60 s, 90 s and 120 s) and motor condition (walking or sitting) as fixed effects. We also included the variable subjects as random effects and the variable sex as covariate. The *p*-values obtained from post hoc analyses were adjusted using the Bonferroni correction.

For detailing the effect of the cognitive task, we also tested whether some estimation biases (averaged over participants in the same task and the same time interval) were not significantly different from zero with one-sample Wilcoxon signed-rank tests.

Then, to investigate how temporal estimation changes as a function of duration, for each condition, data of all participants for all tasks and durations, binned over 2 s, were fitted with a linear function with 2 parameters. χ^2^ were used as goodness-of-fit tests. Z-tests were used to assess whether the fitting curves’ slopes were significantly different from zero and to compare the slopes for each task and condition.

For measuring the dependency of estimation uncertainty on duration, RMSE values, binned over 2 s durations, were fitted with 1-parameter linear functions, and χ^2^ were used as goodness-of-fit tests. To statistically compare the trend of RMSE in each task and condition, the slopes and intercepts of the fitting curves were compared with z-tests.

Then, to evaluate whether our data follow the scalar timing theory, RMSE values were fitted as a function of estimated time with 1-parameter linear functions, and χ^2^ were used as goodness-of-fit tests. The coefficients of variation for each task and condition were compared with the z-tests.

The averaged percentages of correct responses and response times for the solve simple and solve hard tasks in the two motor conditions were compared with two-way ANOVA analysis, with factor condition (two levels: sitting vs. walking) and time interval (five levels: 15 s, 30 s, 60 s, 90 s, 120 s).

Finally, to exclude the possibility that results could depend on physical fatigue, mental tiredness and temperature, all factors that are known to affect time estimation, we tested the correlations of these parameters with the block number with regression model fits. R-squared were used as goodness-of-fit tests. T-statistics assessed the significant increase/decrease in these variables as a function of block number (by testing whether the angular coefficient of the fitting curve was different from zero). The same analyses were carried out to exclude the effect of block number on time estimation itself. Particularly, we checked that percentage time estimation difference in the look and solve hard tasks (averaged across all time intervals) did not change as a function of block number.

Matlab (R2020b version) and Excel (16 version) software were used for data processing and graph creation. Data fitting was performed with Mathematica software (Wolfram). R (4.0.3 version) and JASP (Version 0.8.6) software were used for statistical analyses.

## 3. Results

Mixed-effects linear model analysis revealed the main effect of cognitive task (χ^2^(3) = 2265.5, *p* < 0.001), time interval (χ^2^(4) = 991.6, *p* < 0.001), motor condition (χ^2^(1) = 41.0, *p* < 0.001) and their interaction (χ^2^(12) = 21.3, *p* < 0.05), on time estimation. No significant differences were found as a function of sex (χ^2^(1) = 2.07, *p* > 0.05). In the next paragraphs, we will investigate in detail the effects of these three main factors.

### 3.1. Effects of Cognitive Task on Time Estimation

**Figure 3:**
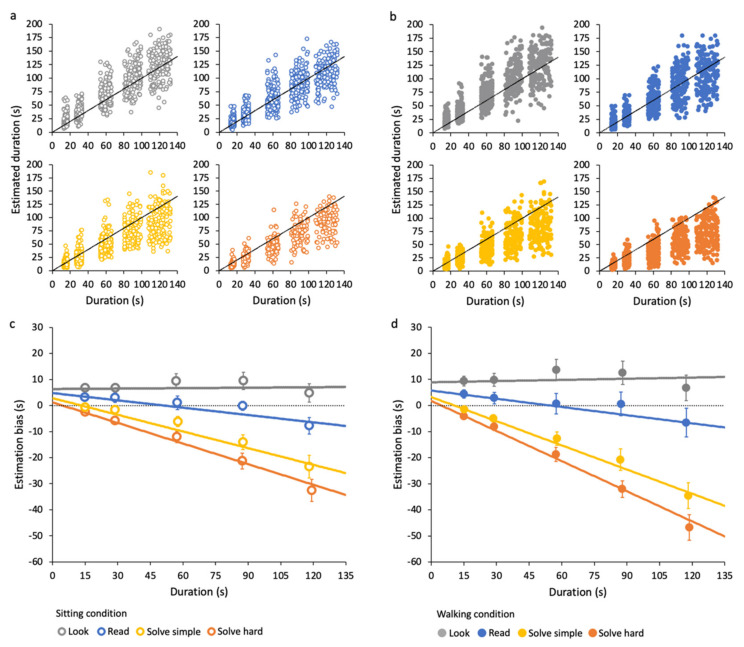
Time estimations during different tasks. (**a**) Sitting condition: different panels represent all data in different tasks (see different colors in the legend). (**b**) Walking condition: different panels represent all data in different tasks (see different colors in the legend). (**c**) Sitting condition: estimation bias averaged over time intervals and participants. The dashed lines represent best fits calculated on data averaged on smaller time intervals (2 s, see text). Look: intercept = 6.4 ± 0.9, slope = 0.005 ± 0.2, χ^2^ (35) = 45.9. Read: intercept = 4.8 ± 0.8, slope = −0.09 ± 0.2, χ^2^ (35) = 50.1. Solve simple: intercept = 2.8 ± 0.6, slope = −0.2 ± 0.2, χ^2^ (35) = 57.8. Solve hard: intercept = 1.2 ± 0.4, slope = −0.3 ± 0.01, χ^2^ (35) = 36.1 (**d**) Walking condition: estimation bias averaged over time intervals and participants. Solid lines represent best-fit curves, calculated as in c). Look: intercept = 8.9 ± 0.8, slope = 0.01 ± 0.2, χ^2^ (35) = 35.6. Read: intercept = 5.7 ± 0.8, slope = −0.1 ± 0.2, χ^2^ (35) = 41.6. Solve simple: intercept = 3.3 ± 0.6, slope = −0.3 ± 0.2, χ^2^ (35) = 40. Solve hard: intercept = 1.8 ± 0.4, slope = −0.4 ± 0.01, χ^2^ (35) = 36.7. Error bars are SE across participants.

Raw data of all participants for the different types of tasks in the sitting and the walking condition are reported in Figure 3a,b, respectively. For the large majority of time intervals, data are not normally distributed (see Supplementary Material—Figure S1 and Table S1). Regardless of the motor condition, data clearly show that participants tend to overestimate time if they are not required to perform any particular task (look task, see Figure 3a,b—first panel). They report a more or less accurate estimation while they are reading (read task, see Figure 3a,b—second panel). They mostly underestimate durations if they are engaging in demanding tasks, with increasing underestimation for harder tasks (solve simple and solve hard tasks, see Figure 3a,b—third and fourth panels). Estimations averaged across all participants for each time interval are reported in Table 1.

Differences between effective and estimated durations (estimation bias), averaged across participants, for each task and time interval, are reported in Figure 3c,d for the sitting and walking conditions, respectively. All pairwise post hoc comparisons across average estimations in each task for each time interval are reported in Supplementary Material—Table S2.

Temporal estimation in some combinations of task and time interval is very accurate. In fact, in some cases, the estimation bias is not significantly different from zero (results of one-sample Wilcoxon signed-rank test are reported in Supplementary Material—Table S3). Overall, participants give the most accurate estimation while reading, even if not for all the tested time intervals. During other tasks, estimation accuracy greatly changes based on the duration. For this reason, in the next section we will describe the effects of duration on the estimation bias in each task (represented by the fitting curves in Figure 3c,d, see Section 2.4 for details).

### 3.2. Effects of Duration on Time Estimation

All pairwise post hoc comparisons across average estimations of different time intervals for each task, in the sitting and walking condition, are reported in Supplementary Material—Table S4.

Results show that estimation bias depends on duration in a different way for each cognitive task. Since data are not normally distributed within the time intervals considered (see Supplementary Material—Figure S1 and Table S1), they were averaged over 2 s time intervals for linear fitting purposes (see Section 2.4). For clarity, however, fitting curves are shown together with data averaged over the unbinned time intervals in Figure 3a,b for the sitting and walking condition, respectively. Fit parameters and goodness-of-fit values are reported in the figure caption.

In the look task, the estimation bias does not vary with time, both in the sitting and walking conditions; that is, slopes are not statistically different from 0 (sitting: z = 0.3, *p* > 0.05; walking: z = 0.8, *p* > 0.5). In the read task, in both motor conditions, the estimation bias becomes slightly more negative (increases in absolute value), going from shorter to longer durations; that is, slopes are both statistically different from 0 (sitting: z = −5.3, *p* < 0.001; walking: z = −5, *p* < 0.001). In the two solve tasks, the fitting curves have steep negative slopes, all significantly different from zero (solve simple sitting z = −11.5, *p* < 0.001; solve simple walking z = −16.7, *p* < 0.001; solve hard sitting z = −19.5, *p* < 0.001; solve hard walking z = −27.1, *p* < 0.001). The slopes of look, read and solve tasks are significantly different from each other (all z-tests results are reported in Supplementary Material—Table S5). Additionally, the slopes for solve hard task are more negative than those for solve simple, indicating that time underestimation while performing cognitive-demanding tasks increases with task difficulty. Note that estimation biases (Figure 3c,d) have the same trends in both motor conditions, although they differ in magnitude, as will be described in the next section.

**Figure 4:**
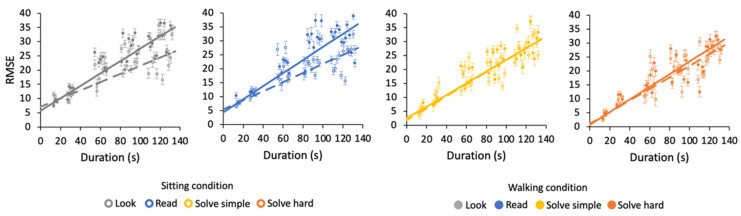
Estimation uncertainty as a function of duration. Averaged RMSE computed on 2 s intervals for all tasks and conditions with their best-fit curves. Different panels correspond to different tasks (see colors in the legend). Sitting condition: open symbols—dashed lines; walking condition: solid symbols—solid lines. Sitting condition—Look: slope = 0.14 ± 0.02, intercept = 4.6 + 0.2; Read: slope = 0.16 ± 0.01, intercept = 4.1 + 0.4; Solve simple: slope = 0.22 ± 0.02, intercept = 1.7 + 0.1; Solve hard: slope = 0.21 ± 0.01, intercept = 0.8 + 0.1. Walking condition—Look: slope = 0.22 ± 0.1, intercept = 4.9 + 0.1; Read: slope = 0.23 ± 0.01, intercept = 3.2 + 0.2; Solve simple: slope = 0.21 ± 0.01, intercept = 1.2 + 0.1; Solve hard: slope = 0.23 ± 0.02, intercept = 0.9 + 0.2.

To shed some light on the mechanisms underlying time estimation, we measured uncertainties for different durations in all tasks and motor conditions. Figure 4 shows root mean square errors (RMSE) calculated over 2 s bins for linear fitting purposes (see Section 2.4). In each task and condition, we found RMSE to increase with duration. In the sitting condition, the variabilities associated with different tasks are comparable (see caption of Figure 4). In the walking condition, the variabilities associated with low-demanding look and read tasks are higher than those for high-demanding solve tasks (see caption of Figure 4). Comparisons of fitting curves in the walking and sitting condition are described in the next section.

**Figure 5:**
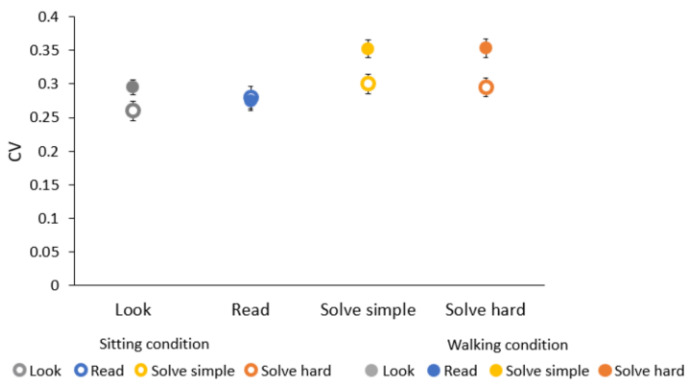
Coefficient of variation for different tasks in sitting and walking conditions. Data shown and their errors are the results of best linear fitting of binned RMSE as a function of estimated time.

We also fitted RMSE as a function of estimated time (not shown here). Coefficients of variation (CV) for each task and condition (slopes of fitted data) are reported in Figure 5. Results show that CV in the sitting condition are statistically the same for all tasks. Instead, in the walking condition, CV for solve simple and solve hard tasks are higher than those for look (z = −3.0, *p* < 0.01; z = −2.0, *p* < 0.05, respectively) and read tasks (z = −2.7, *p* < 0.01; z = −2.7, *p* < 0.01, respectively).

### 3.3. Effects of Walking on Time Estimation

Another interesting result emerging from our data is the difference in time estimation between the walking and the sitting conditions.

**Figure 6:**
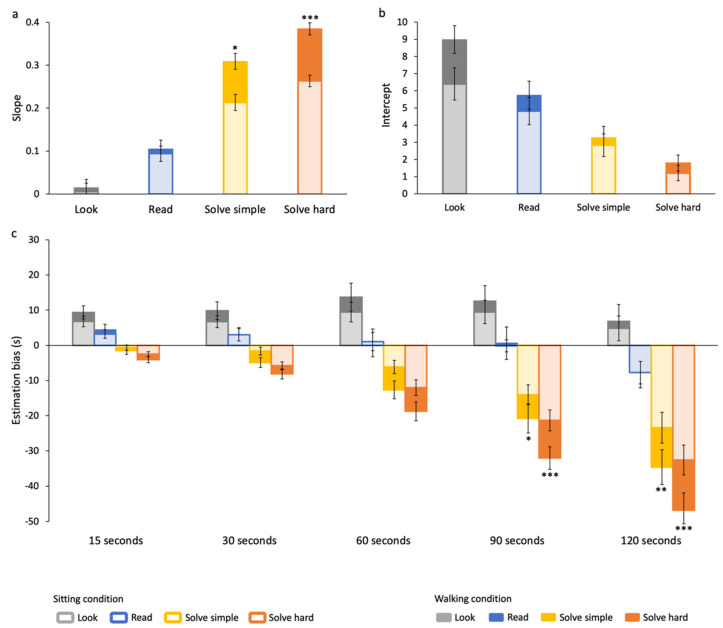
Comparison of time estimation during walking and while sitting for all tasks. (**a**) Slopes of best-fit lines, shown in Figure 3c,d. Asterisks mark statistically significant differences with z-tests: * *p* < 0.05, *** *p* < 0.001. (**b**) Y-axis intercepts of best-fit lines, shown in Figure 3c,d. (**c**) Estimation bias averaged over time intervals and participants. Asterisks mark statistically significant pairwise post hoc comparisons: * *p* < 0.05, ** *p* < 0.01, *** *p* < 0.001.

To analyze the overall effect of walking on time estimation accuracy during different tasks, we compared the slopes (Figure 6a) and the intercepts (Figure 6b) of the data fitting curves in the two motor conditions. In the two solve tasks, the slopes are higher in the walking than in the sitting condition (solve simple: z = 2.5, *p* < 0.05; solve hard: z = 4, *p* < 0.001). This means that during walking, a larger time underestimation is observed, particularly during demanding tasks. The intercepts, representing a constant error in estimation, are also larger in the walking than in the sitting condition in all tasks, although not to a statistically significant level.

Estimation biases averaged across participants for each task and time interval in the two motors conditions are reported in Figure 6c to highlight the effects of walking at each time interval. All pairwise post hoc comparisons across average estimations in the sitting and walking condition are reported in Supplementary Material—Table S6 (significant differences are represented by asterisks in Figure 6c). Results show that estimation during walking is less accurate within each cognitive task, but the direction of the error differs across them. Participants’ overestimation for the look task tends to be larger during walking compared to sitting for all time intervals, although it is not statistically significant. For the read task, the accuracy is comparable between the two conditions in all time intervals. In the solve tasks, walking induces a larger underestimation at all time intervals, significant for the longer durations.

Estimation during walking is also less precise than when sitting, only for low-demanding tasks (see Figure 4). Indeed, RMSE in the walking condition are higher than in the sitting condition in the look (z = −2.3, *p* < 0.05) and read tasks (z = −2.7, *p* < 0.05).

Coefficients of variation for solve tasks in the walking condition are also larger than in the sitting condition (solve simple, z = 2.0, *p* < 0.05; solve hard, z = 2.0, *p* < 0.05) (see Figure 5). The size of the increase is about 15%. There is no difference between CV of the walking and sitting condition for read and look tasks.

To compare the magnitude of the effects induced by the cognitive and motor processing on time estimation, percentual biases (e.g., relative to time interval) were considered. To evaluate the effect of the motor condition per se in our data, differences between estimates in the walking and sitting condition in the look task, not requiring cognitive efforts, have to be considered. An average effect of about 7.5% is found (cf. dark-gray vs. light-gray bars in Figure 6), with a maximum effect of about 17% at 15 s. To evaluate the effect of cognitive tasks per se, differences between estimates in the solve and look tasks in the sitting condition, not requiring motor processing, have to be considered. The average effect is about 37% (average difference at all time intervals of light-yellow and light-orange bars vs. averages of light-gray bars in Figure 6). The maximum influence of cognitive tasks is about 62%, corresponding to the difference between solve hard and look task at 15 s time interval (cf. light-gray vs. light-orange bars in Figure 6).

### 3.4. Effects of Walking on Cognitive Performance

Additional results emerge from the comparison of participants’ performance in those tasks requiring the solving of mathematical operations of different difficulties (solve simple and solve hard tasks), in the sitting and walking condition.

**Figure 7:**
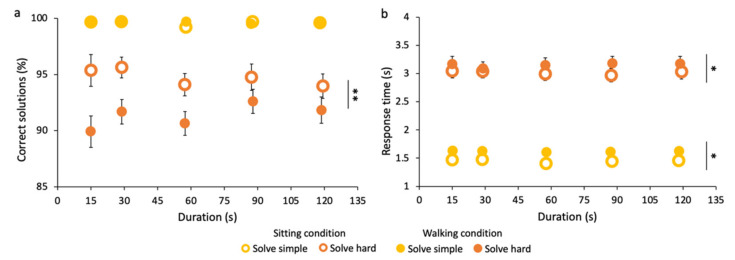
Performance in the math tasks while walking and sitting. (**a**) Percentage of correct solutions. (**b**) Response time to operations. Responses for correct and incorrect solutions are considered. Asterisks mark statistically significant differences with ANOVAs: * *p* < 0.05, ** *p* < 0.01. Error bars are SE across participants.

First, the percentages of correct solutions for mathematical operations differ across the two tasks and conditions but not across time intervals (see Figure 7a). Indeed, as expected, participants gave a higher number of correct answers to easier operations than to harder ones, in both motor conditions. Averaged across all durations, in the sitting condition, the averaged percentage of correct responses is 99.6 ± 0.2% for simple sums and 94.7 ± 1.1% for hard sums, while in the walking condition, it is 99.6 ± 0.1% for simple sums, and 91.3 ± 1.2 for hard sums. Only in the solve hard task does the main effect of motor condition emerge (F(1,15) = 11.9, *p* < 0.01, η^2^ = 0.1). Thus, the percentage of correct responses to simple operations is the same in the two conditions, possibly due to ceiling effects. Instead, the performance for hard operations is significantly lower in the walking than in the sitting condition for all time intervals, with an average decrease of 4%.

Differences between the two tasks and the two conditions also emerge when considering the averaged time needed by participants to solve the operations (see Figure 7b). Again, as expected, in both conditions, participants solve the easier operations faster than the harder ones. In the sitting condition, the averaged response time is 1.45 ± 0.05 s for simple sums and 3.02 ± 0.1 s for hard sums, while in the walking condition, it is 1.62 ± 0.04 s for simple sums and 3.16 ± 0.1 s for hard sums. For both tasks, there is the main effect of condition (solve simple: F(1,15) = 6.1, *p* < 0.5, η^2^ = 0.2; solve hard: F(1,15) = 4.7, *p* < 0.05, η^2^ = 0.03) but not of time intervals and their interactions. Thus, the average time necessary to solve simple and hard sums is significantly lower in the sitting than in the walking condition. Interestingly, the average time differences between walking and sitting conditions for solve simple and solve hard, respectively, 0.17 and 0.14 s, are statistically comparable (paired-sample *t*-test: t(15) = 0.35, *p* > 0.5); that is, walking affects response times in the same way, independently of task difficulty.

Overall, our results suggest that performing a motor task impairs the performance in a concurring cognitive task and slows down response times.

### 3.5. Controls of Influencing Variables: Physical Fatigue, Mental Tiredness and Body Temperature

Since participants were subjected to a long, demanding experiment, it was likely that, despite the frequent breaks, they became increasingly tired with the succession of blocks. To check for this, after each block, participants were asked to rate their physical fatigue with a 7-point Likert scale (see Supplementary Material—Figure S2a). On average, for the sitting condition, the minimum score reported was 1.4 ± 0.1, and the maximum was 1.7 ± 0.1, with an increase of 0.3 from block 1 to 10 (averaged across the two sessions). For the walking condition, the minimum physical fatigue score was 1.7 ± 0.2, and the maximum was 3.3 ± 0.3, with an increase of 1.6 from block 1 to 10. Regression model fits were performed to investigate the relationship between physical fatigue and block number. As expected, in the sitting condition, physical fatigue does not increase with the block number (b = 0.01, t(8) = 2.1, *p* > 0.05; see Figure S2a—left panel). In the walking condition, however, the subject’s fatigue significantly increases with blocks, that is, with the walking time (b = 0.15, t(8) = 6.9, *p* < 0.001; see Figure S2a—right panel).

Since the experiment included trials requiring participants to solve mathematical operations, the whole data collection was also highly cognitive demanding for the participants. For this reason, they were asked to rate their mental tiredness after each block (see Supplementary Material—Figure S2b). On average, for the sitting condition, the minimum score was 1.7 ± 0.1, and the maximum was 2.9 ± 0.2, with an increase of 1.2 from block 1 to 10 (averaged across the two sessions). For the walking condition, the minimum score was 1.9 ± 0.2, and the maximum was 3.4 ± 0.2, with an increase of 1.5 from block 1 to 10. Regression model fits showed that mental tiredness significantly increases with the succession of blocks, both for the sitting (b = 0.13, t(8) = 6.9, *p* < 0.001; see Figure S2b—left panel) and the walking condition (b = 0.16, t(8) = 8.1, *p* < 0.001; see Figure S2b—right panel).

Since physical and mental fatigue actually change from the beginning to the end of the experimental sessions, we performed further analyses to check that their increase did not influence time estimation across blocks (see Supplementary Material—Figure S2c). To do this, we evaluated time estimation differences in the look and solve hard tasks (the easiest and the most difficult tasks) from block 1 to 10, averaged across all participants, durations and the two sessions. Linear regression analysis shows that time estimation difference for both tasks and both conditions does not change as a function of block number (all t-statistics yield *p* > 0.05).

Furthermore, the experimenter measured participants’ temperature at the beginning of each trial, to check it remained stable during the whole data collection (data reported in Supplementary Material—Figure S2d). Averaged across the two sessions, the medium temperature of participants was 36.7 ± 0.01 °C in the sitting condition and 36.5 ± 0.02 °C in the walking condition. Regression analysis confirms that body temperature does not increase during the experiment, neither in the sitting (b = 0.002, t(8) = 0.9, *p* > 0.05; see Figure S2d—left panel) nor in the walking condition (b = 0.0003, t(8) = 0.06, *p* > 0.05; see Figure S2d—right panel).

These analyses indicate that our results for the different cognitive tasks, time intervals and motor conditions must depend on cognitive and motor processes alone, which influence accuracy and precision of time estimation.

## 4. Discussion

In the present study, the ability of human participants to estimate time while concurrently performing cognitive and motor tasks was investigated for the first time. In each trial, participants were randomly required to perform a cognitive task of variable difficulty (look at the screen, read aloud some numbers, solve easy or hard mathematical sums) and estimate the duration elapsed from the beginning to the end of the trial. The effects of gross body movements on time estimation were investigated here by measuring verbal prospective estimation in two different motor conditions: sitting on a chair or walking on a treadmill. Here, we tested the prospective estimation of durations of up to two minutes (specifically from 13 to 132 s). While durations below a second have received a great deal of attention in the human timing literature, a lower number of studies tested the effects of cognitive tasks on such long intervals ([49,61], for review, see Refs. [12,13,37,43]). However, in ecological situations, human activities require more than just a few seconds, and we usually do have to estimate time in terms of minutes and even more while performing other tasks. Additionally, by using such an extended time interval, we were able to measure how estimation accuracy changes across durations, allowing us to test quantitatively the time estimation mechanisms underlying different concurring mental tasks.

First, when participants are not involved in any particular cognitive task, such as simply looking at a blank screen (“*look* task”), they tend to overestimate temporal intervals, in agreement with previous works [42,49,68]. Time overestimation could depend on the fact that paying attention only to time lengthens subjective durations [37,39,40,41,45]. The bias as a function of durations remains constant, suggesting a fixed shift in time estimation. Polti et al., 2010 (see Ref. [49], Figure 2), using the same paradigm and task, and similar long durations, found data consistent with our effect. In the *look* task, time overestimation tends to be higher in the walking condition, as in previous studies [57,58], although the effect is not statistically significant. Estimation bias as a function of duration is flat, as in the sitting condition. Estimation uncertainty is also lower in the walking condition, meaning that there is higher variability during walking.

When participants read numbers presented on the screen (“*read* task”), their accuracy is quite high, especially for intervals of intermediate duration. Indeed, subjective durations are compatible with the effective elapsed duration for 30 to 90 s time intervals. Estimation bias shows that there is a modest overestimation for the briefest durations, which decreases with time, becoming a modest underestimation for the longest durations. Overall, these results confirm that when participants are engaged in intermediately demanding cognitive tasks, such as reading, they are more accurate in time estimation compared to easier or more difficult tasks [49]. These effects could also be accounted for by the allocation of attention. It might be that the reading activity is not demanding enough to fully deviate attention away from time (which would cause time underestimation), but, at the same time, it prevents subjects from only paying attention to time (which would cause time overestimation). This interpretation would also explain the small overestimation at 15 s and the small underestimation observed around 120 s, since participants might still be able to concentrate on a temporal task for a few seconds, but then, the succession of numbers to be read progressively diverts the subject’s attention from the temporal task. In this cognitive task, the effect of walking is less prominent than in all other tasks, being non-existent in the estimation accuracy and only emerging in estimation uncertainty (higher variability during walking).

Time estimation is completely different while participants are solving mathematical operations of low or high difficulty (“*solve* simple” and “*solve* hard” tasks). They tend to underestimate temporal durations with greater magnitude in the most difficult task. These effects are in line with the results usually found with prospective estimation paradigms, as the one used here, in which cognitive effort induces duration underestimation [49]. Underestimation while participants are engaged in cognitive-demanding task might also be explained by the hypothesis that diverting attention away from time shortens subjective duration [37,39,40,41,45]. The estimation bias progressively increases as a function of duration, and the increase is more pronounced for the harder task. Furthermore, estimation uncertainty increases with duration in a way that is consistent with an underlying mechanism with fixed relative uncertainty on time estimation.

Very interestingly, when participants perform the math operations while walking, they underestimate temporal durations more than they already do while they are seated. Moreover, the increase in bias with increasing duration is more pronounced in the walking condition. Contrary to low-demanding tasks, there are no differences in variability between the two motor conditions. There are no existing hypotheses for explaining these results, given our novel experimental paradigm of concurrent motor and non-temporal cognitive-demanding tasks while performing time estimation. We can speculate that the higher time distortion might be due to the fact that the cognitive effort spent to perform the motor task adds to that needed to perform the cognitive one. The hypothesis that motor and cognitive processing interact is in line with the studies showing that the motor task induces an impairment on performance in the mental task [50,51]. We tested this hypothesis and found interference in cognitive performance. While walking, participants provided a larger number of wrong solutions to the hard mathematical operations and needed more time to provide the answer to the easy and the hard operations compared to sitting. Thus, this cognitive–motor interaction could also be reflected in time estimation distortions.

The time estimation effects observed in this study do not depend on biological variables, such as body temperature or physical and mental fatigue. The latter slightly increased during each experimental session, as reported subjectively, but never reached levels high enough to cause physical and mental stress.

Overall, our results show that both cognitive and motor processes influence time estimation. However, it should be noted that the magnitude of their effect is very different, since the cognitive task has a much higher effect than the motor task (about four times as much) at all time intervals.

The representation of time in the brain is still under investigation, but there is general agreement on the fact that time perception processes do not rely on a specific brain structure but rather on a broad network of brain areas [69,70,71]. Particularly, studies indicate the role of basal ganglia, supplementary motor area, frontal lobes and the cerebellum, which are also in charge of cognitive and sensorimotor functions (e.g., walking) [70,71,72]. Moreover, the right parietal cortex, which is consistently implicated in mathematical cognition [73], has also been found to be crucial in time estimation [74]. Therefore, we can speculate that the interference effects we found in our study reflect the neural interaction between cognitive, motor and time perception functions, which share some neural resources.

## 5. Conclusions

We conclude that time estimation is influenced by both cognitive and motor tasks. The effects found in our study could be framed within the widespread pacemaker accumulator model, which assumes the presence of an internal clock sending pulses to an accumulator to keep track of the elapsed time [2,3,4]. In this framework, attention acts as a switch that alters the number of pulses transferred to the accumulator and then to working memory (attentional allocation model, [5,12,13,14]). Our results—temporal overestimation while fully attending to time and temporal underestimation during cognitive-demanding tasks—are in line with this model. In fact, when attention is diverted from just the passage of time by a cognitive task, the switch is opened and leads to the loss of a certain number of pulses, thus decreasing the perceived duration. In every task, the variability increases linearly with time estimates, in agreement with the broadly demonstrated scalar timing theory [3,13,14]. It is also notable that our cognitive task, while causing an underestimation, does not increase the relative variability of the estimate, indicating that the amount of lost time is relatively constant from trial to trial.

The additional underestimation induced by simultaneous motor and cognitive tasks is harder to frame in the literature, since no previous studies combined motor, mental and temporal tasks in a single paradigm. It is also interesting that the same motor task by itself does not induce significant changes in time perception (except possibly for a slight extra bias), so the two effects do not simply add linearly. We can speculate that the motor task increases somehow the weight of the concurrent cognitive effort, leading to additional openings of the switch and, consequently, to a lower number of accumulated pulses than in the sitting condition. This reflects also in the relative uncertainty (coefficients of variation), which is higher in the walking than in the sitting condition. The additional cognitive effort could be provided by irregular walking speed used in our paradigm, which requires participants to continuously adapt to the random change of speed, making the deambulatory activity non-automatic [75]. It would be interesting to test this hypothesis by using different paradigms, including regular constant walking speed or even more pronounced speed changes.

## Figures and Tables

**Table 1:** Time estimation averaged over every participant for each task and time interval (with standard errors across participants), in the sitting and walking conditions.

Sitting	15 s	30 s	60 s	90 s	120 s
Look	M = 21.8, SE = 1.5	M = 35.6, SE = 1.7	M = 66.3, SE = 2.8	M = 97.3, SE = 3.2	M = 122.9, SE = 3.4
Read	M = 18.1, SE = 1.2	M = 31.8, SE = 1.7	M = 58.5, SE = 2.4	M = 87.3, SE = 1.7	M = 110.3, SE = 3.4
Solve simple	M = 14.4, SE = 0.7	M = 27.1, SE = 1.1	M = 51.5, SE = 1.7	M = 73.6, SE = 2.8	M = 94.7, SE = 4.3
Solve hard	M = 12.7, SE = 0.7	M = 23.1, SE = 1.1	M = 45.2, SE = 2.2	M = 65.9, SE = 2.9	M = 86.7, SE = 3.9
Walking	**15 s**	**30 s**	**60 s**	**90 s**	**120 s**
Look	M = 24.4, SE = 1.8	M = 38.7, SE = 2.4	M = 71.2, SE = 3.8	M = 100.6, SE = 4.5	M = 124, SE = 4.7
Read	M = 19.4, SE = 1.5	M = 31.8, SE = 1.9	M = 58.2, SE = 3.9	M = 87.8, SE = 4.5	M = 110.8, SE = 5.4
Solve simple	M = 13.6, SE = 1.1	M = 23.7, SE = 1.3	M = 45.1, SE = 2.5	M = 66.2, SE = 4.1	M = 83.7, SE = 4.7
Solve hard	M = 10.9, SE = 0.8	M = 20.8, SE = 1.4	M = 38.6, SE = 2.6	M = 55.9, SE = 3.1	M = 71.9, SE = 4.6

## Data Availability

All data are available from the Zenodo database (DOI: 10.5281/zenodo.6107737).

## Supplementary Materials

The following supporting information can be downloaded at: https://www.mdpi.com/article/10.3390/brainsci12030404/s1, Figure S1. Data distributions for each task and time interval; Figure S2. Biological variables and time estimation as a function of block number; Table S1. Results of Shapiro–Wilk test for normality; Table S2. Post hoc comparisons across cognitive tasks for each time interval in the sitting and walking conditions; Table S3. Results of one-sample Wilcoxon signed-rank test; Table S4. Post hoc comparisons across durations for each cognitive task in the sitting and walking conditions. Table S5. Results of z-tests between the slopes of best-fit lines, shown in Figure 3c,d; Table S6. Post hoc comparisons between motor conditions for each task and duration.
