# Supplementary material for: Influence of Motor and Cognitive Tasks on Time Estimation
Source: Brain Sci. 2022 Mar 18;12(3):404. doi: 10.3390/brainsci12030404 (PMC8946194; doi:10.3390/brainsci12030404)
Supplement: Supplementary file 1 [file brainsci-12-00404-s001.zip › brainsci-1574685-supplementary.pdf]

# Supplementary Material

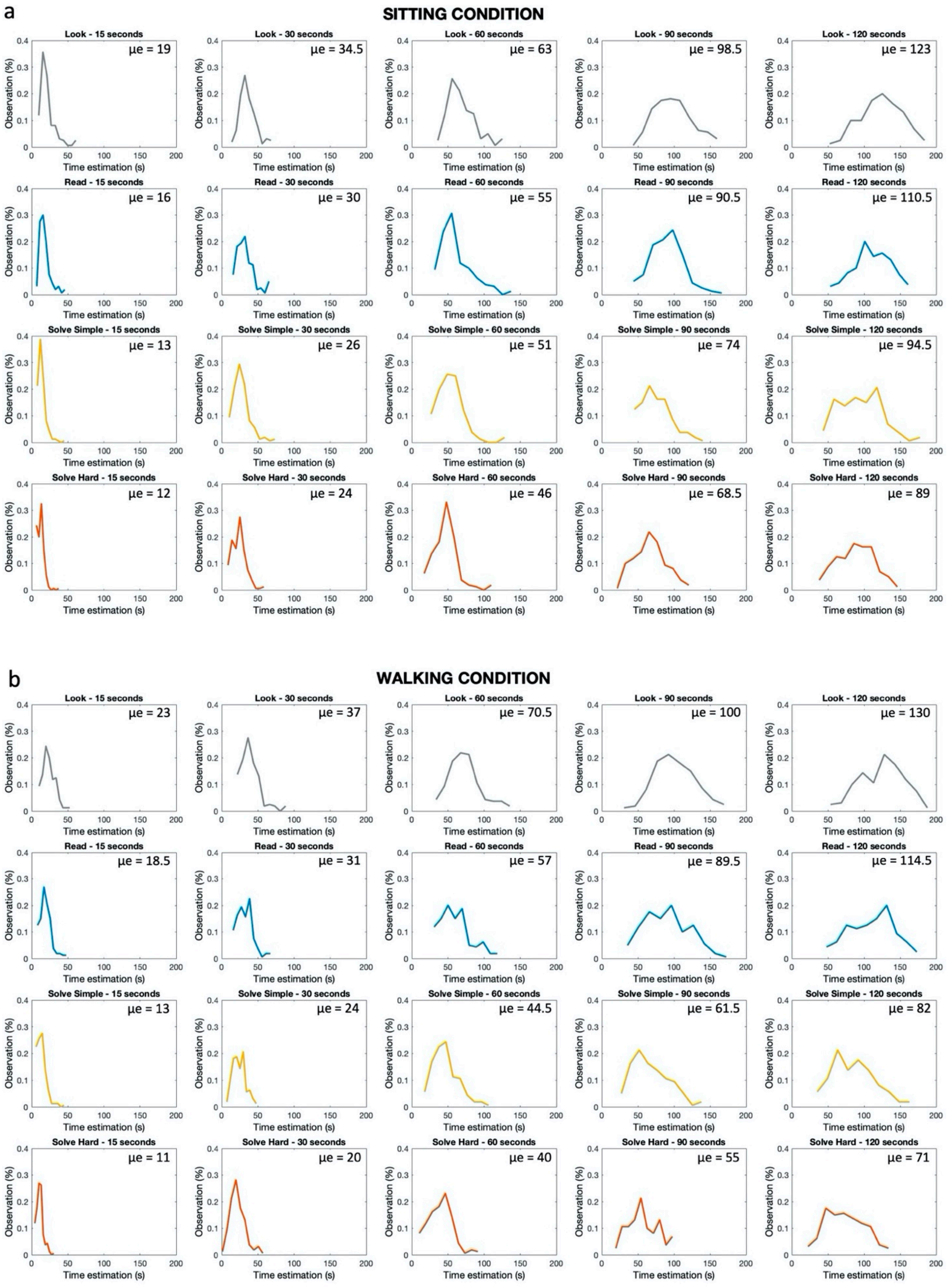

**Figure S1. Data distributions for each task and time interval. (a) Sitting condition. (b) Walking condition.** The medians of the distributions are reported over each graph.

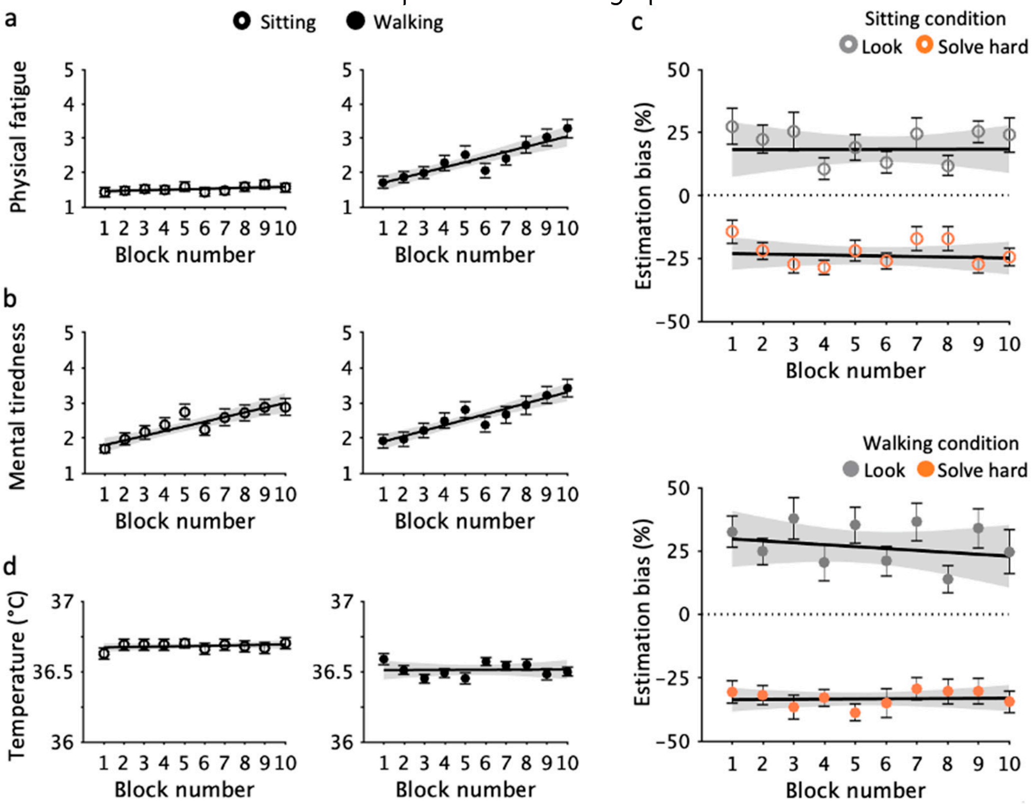


**Figure S2. Biological variables and time estimation as a function of block number. (a) Physical fatigue.** Medium scores of participants. Error bars represent SE. In the sitting condition (left panel), physical fatigue remains stable across blocks ($b = .01$, $t(8) = 2.1$, $p > .05$); in the walking condition (right panel) fatigue significantly increases with block number ($b = .15$, $t(8) = 6.9$, $p < .001$; $R^2 = .86$, $F(8) = 48.8$, $p < .001$). **(b) Mental tiredness.** Medium scores of participants. Error bars represent SE. In the sitting condition (left panel) mental tiredness significantly increases with block number ($b = .13$, $t(8) = 6.9$, $p < .001$; $R^2 = .86$, $F(8) = 47.8$, $p < .001$), as well as in the walking condition (right panel) ($b = .16$, $t(8) = 8.1$ , $p < .001$; $R^2 = .89$, $F(8) = 66.2$, $p < .001$). **(c) Time estimation.** Percentage estimation bias in the *look* and *solve hard* tasks, averaged across time intervals, does not change as a function of block number, neither in the sitting (upper panel) or the walking condition (lower panel). **(d) Temperature.** Body temperatures averaged across participants and SE. Participants' temperature does not change as a function of block number, neither in the sitting (left panel) or the walking condition (right panel). Shaded areas represent 95% confidence intervals.

**Table S1. Results of Shapiro Wilk test for normality.** P-values below 0.05 indicate that the data significantly deviate from a normal distribution.

| Sitting | 15 seconds | 30 seconds | 60 seconds | 90 seconds | 120 seconds |
|---|---|---|---|---|---|
| *Look* | W=0.82, p < 0.001 | W=0.95, p < 0.001 | W=0.94, p < 0.001 | W=0.98, p < 0.05 | W=0.99, p > 0.05 |
| *Read* | W=0.85, p < 0.001 | W=0.92, p < 0.001 | W=0.92, p < 0.001 | W=0.99, p > 0.05 | W=0.99, p > 0.05 |
| *Solve simple* | W=0.85, p < 0.001 | W=0.91, p < 0.001 | W=0.91, p < 0.001 | W=0.96, p < 0.001 | W=0.97, p < 0.01 |
| *Solve hard* | W=0.91, p < 0.001 | W=0.95, p < 0.001 | W=0.93, p < 0.001 | W=0.98, p < 0.05 | W=0.99, p < 0.001 |

| Walking | 15 seconds | 30 seconds | 60 seconds | 90 seconds | 120 seconds |
|---|---|---|---|---|---|
| *Look* | W=0.96, p < 0.001 | W=0.91, p < 0.001 | W=0.96, p < 0.001 | W=0.99, p > 0.05 | W=0.98, p > 0.05 |

| | | | | | |
|---|---|---|---|---|---|
| *Read* | W=0.92, p < 0.001 | W=0.96, p < 0.001 | W=0.95, p < 0.001 | W=0.98, p > 0.05 | W=0.98, p > 0.05 |
| *Solve simple* | W=0.88, p < 0.001 | W=0.97, p < 0.01 | W=0.96, p < 0.001 | W=0.97, p < 0.01 | W=0.97, p < 0.01 |
| *Solve hard* | W=0.92, p < 0.001 | W=0.95, p < 0.001 | W=0.98, p < 0.05 | W=0.97, p < 0.01 | W=0.98, p < 0.05 |

**Table S2. Post-hoc comparisons across cognitive tasks for each time interval in the sitting and walking conditions.**

| Sitting | *Read* | *Solve simple* | *Solve hard* | *Read* | *Solve simple* | *Solve hard* |
|---|---|---|---|---|---|---|
| | | **15 seconds** | | | **30 seconds** | |
| *Look* | t=3.6, p>0.05 | t=7.8, p>0.05 | t=9.6, p<0.05 | t=3.7, p>0.05 | t=8.7, p<0.05 | t=13.2, p<0.001 |
| *Read* | - | t=4.1, p>0.05 | t=5.9, p>0.05 | - | t=4.9, p>0.05 | t=9.5, p<0.01 |
| *Solve simple* | - | - | t=-1.0 p>0.05 | - | - | t=-4.5, p>0.05 |
| | | **60 seconds** | | | **90 seconds** | |
| *Look* | t=9.5, p<0.05 | t=17.1, p<0.001 | t=23.7, p<0.001 | t=9.9, p<0.01 | t=17.6, p<0.001 | t=24.1, p<0.001 |
| *Read* | - | t=-6.9, p>0.05 | t=14.1, p<0.001 | - | t=7.6, p>0.05 | t=13.6, p<0.001 |
| *Solve simple* | - | - | t=-6.5, p>0.05 | - | - | t=-14.6, p<0.001 |
| | | **120 seconds** | | | | |
| *Look* | t=13.4, p<0.001 | t=30.2, p<0.001 | t=38.1, p<0.001 | | | |
| *Read* | - | t=7.1, p>0.05 | t=24.7, p<0.001 | | | |
| *Solve simple* | - | - | t=-7.9, p>0.05 | | | |

| Walking | *Read* | *Solve simple* | *Solve hard* | *Read* | *Solve simple* | *Solve hard* |
|---|---|---|---|---|---|---|
| | | **15 seconds** | | | **30 seconds** | |
| *Look* | t=4.9, p>0.05 | t=10.8, p<0.01 | t=13.4, p<0.001 | t=7.1, p>0.05 | t=15.2, p<0.001 | t=18.1, p<0.001 |
| *Read* | - | t=5.8, p>0.05 | t=8.4, p<0.05 | - | t=8.1, p>0.05 | t=10.9, p<0.001 |
| *Solve simple* | - | - | t=-2.6, p>0.05 | - | - | t=-2.9, p>0.05 |
| | | **60 seconds** | | | **90 seconds** | |
| *Look* | t=13.6, p<0.001 | t=27.5, p<0.001 | t=33.8, p<0.001 | t=11.5, p<0.001 | t=33.8, p<0.001 | t=44.9, p<0.001 |
| *Read* | - | t=13.8, p<0.001 | t=20.2, p<0.001 | - | t=22.4, p<0.001 | t=33.4, p<0.001 |
| *Solve simple* | - | - | t=-6.3, p>0.05 | - | - | t=-11.0, p<0.01 |
| | | **120 seconds** | | | | |
| *Look* | t=14.1, p<0.001 | t=41.5, p<0.001 | t=53.2, p<0.001 | | | |
| *Read* | - | t=27.4, p<0.001 | t=39.2, p<0.001 | | | |
| *Solve simple* | - | - | t=-11.7, p<0.001 | | | |

**Table S3. Results of One-Sample Wilcoxon Signed-Rank test.** P-values above 0.05 indicate that the median is not different from 0.

| Sitting | 15 seconds | 30 seconds | 60 seconds | 90 seconds | 120 seconds |
|---|---|---|---|---|---|
| *Look* | Z(15)=133, p < 0.001 | Z(15)=136, p < 0.001 | Z(15)=121, p < 0.01 | Z(15)=111, p < 0.05 | Z(15)=93, p > 0.05 |
| *Read* | Z(15)=111, p < 0.05 | Z(15)=85, p > 0.05 | Z(15)=70, p > 0.05 | Z(15)=58, p > 0.05 | Z(15)=27, p < 0.05 |
| *Solve simple* | Z(15)=52, p > 0.05 | Z(15)=39, p > 0.05 | Z(15)=11, p < 0.01 | Z(15)=0, p < 0.001 | Z(15)=1, p < 0.001 |
| *Solve hard* | Z(15)=10, p < 0.01 | Z(15)=0, p < 0.001 | Z(15)=2, p < 0.001 | Z(15)=0, p < 0.001 | Z(15)=0, p < 0.001 |

| Walking | 15 seconds | 30 seconds | 60 seconds | 90 seconds | 120 seconds |
|---|---|---|---|---|---|
| *Look* | Z(15)=131, p < 0.01 | Z(15)=127, p < 0.01 | Z(15)=124, p < 0.01 | Z(15)=112, p < 0.05 | Z(15)=95, p > 0.05 |
| *Read* | Z(15)=111, p < 0.05 | Z(15)=93, p > 0.0501 | Z(15)=75, p > 0.05 | Z(15)=85, p > 0.05 | Z(15)=46, p > 0.05 |
| *Solve simple* | Z(15)=43, p > 0.05 | Z(15)=8, p < 0.01 | Z(15)=6, p < 0.001 | Z(15)=3, p < 0.001 | Z(15)=0, p < 0.001 |
| *Solve hard* | Z(15)=2, p < 0.001 | Z(15)=4, p < 0.001 | Z(15)=0, p < 0.001 | Z(15)=0, p < 0.001 | Z(15)=0, p < 0.001 |

**Table S4. Post-hoc comparisons across durations for each cognitive task in the sitting and walking conditions.**

| Sitting | *Look* | *Read* | *Solve simple* | *Solve hard* |
|---|---|---|---|---|
| *15s vs 30s* | t=0.4, p>0.05 | t=0.4, p>0.05 | t=1.3, p>0.05 | t=3.9, p>0.05 |
| *15s vs 60s* | t=-3.4, p>0.05 | t=2.4, p>0.05 | t=5.9, p>0.05 | t=10.6, p<0.01 |
| *15s vs 90s* | t=-3.9, p>0.05 | t=2.9, p>0.05 | t=12.7 p<0.001 | t=18.7, p<0.001 |
| *15s vs 120s* | t=-1.8, p>0.05 | t=-11.6, p<0.001 | t=-24.2, p<0.001 | t=-30.3, p<0.001 |
| *30s vs 60s* | t=-3.8, p>0.05 | t=1.9, p>0.05 | t=4.6, p>0.05 | t=6.6, p>0.05 |
| *30s vs 90s* | t=-4.2, p>0.05 | t=2.5, p>0.05 | t=11.4, p<0.001 | t=14.7, p<0.001 |
| *30s vs 120s* | t=-1.4, p>0.05 | t=-11.2, p<0.001 | t=22.9, p<0.001 | t=-26.4, p<0.001 |
| *60s vs 90s* | t=-0.4, p>0.05 | t=0.5, p>0.05 | t=6.8, p>0.05 | t=8.1, p<0.05 |
| *60s vs 120s* | t=-5.3, p>0.05 | t=-9.2, p<0.01 | t=-18.3, p<0.001 | t=-19.7, p<0.001 |
| *90s vs 120s* | t=-5.7, p>0.05 | t=-8.7, p<0.01 | t=-11.5, p<0.001 | t=-11.6, p<0.001 |
| **Walking** | *Look* | *Read* | *Solve simple* | *Solve hard* |
| *15s vs 30s* | t=-0.1, p>0.05 | t=2.0, p>0.05 | t=4.2, p>0.05 | t=4.5, p>0.05 |
| *15s vs 60s* | t=-4.5, p>0.05 | t=4.1, p>0.05 | t=12.5, p<0.001 | t=15.9, p<0.001 |
| *15s vs 90s* | t=-2.7, p>0.05 | t=3.8, p>0.05 | t=20.4 p<0.001 | t=28.8, p<0.001 |
| *15s vs 120s* | t=-3.5, p>0.05 | t=-12.7, p<0.001 | t=-34.3, p<0.001 | t=-43.4, p<0.001 |
| *30s vs 60s* | t=-4.3, p>0.05 | t=2.1, p>0.05 | t=7.9, p>0.05 | t=11.4, p<0.001 |
| *30s vs 90s* | t=-2.5, p>0.05 | t=1.8, p>0.05 | t=16.2, p<0.001 | t=24.2, p<0.001 |
| *30s vs 120s* | t=-3.7, p>0.05 | t=-10.7, p<0.001 | t=-30.1, p<0.001 | t=-38.8, p<0.001 |
| *60s vs 90s* | t=1.8, p>0.05 | t=-0.3, p>0.05 | t=8.2, p<0.05 | t=-12.9, p<0.001 |
| *60s vs 120s* | t=-8.0, p>0.05 | t=-8.8, p<0.05 | t=-22.1, p<0.001 | t=-27.4, p<0.001 |
| *90s vs 120s* | t=-6.2, p>0.05 | t=-8.7, p<0.05 | t=-13.9, p<0.001 | t=-14.5, p<0.001 |

Table S5. Results of *z*-tests between the slopes of best fit lines, shown in Figure 3c and 3d. P-values below 0.05 indicate that the two means are significantly different.

| Sitting | *Read* | *Solve simple* | *Solve hard* |
|---|---|---|---|
| *Look* | z=2.6, p<0.01 | z=5.7, p<0.001 | z=8.1, p<0.001 |

| | *Read* | *Solve simple* | *Solve hard* |
|---|---|---|---|
| *Read* | - | z=3.2, p<0.01 | z=5.4, p<0.001 |
| *Solve simple* | | - | z=1.6, p>0.05 |

| **Walking** | *Read* | *Solve simple* | *Solve hard* |
|---|---|---|---|
| *Look* | z=3.1, p<0.01 | z=8.6, p<0.001 | z=12.2, p<0.001 |
| *Read* | - | z=5.2, p<0.001 | z=7.9, p<0.001 |
| *Solve simple* | | - | z=2.3, p<0.05 |

**Table S6. Post-hoc comparisons between motor conditions for each task and duration.**

| Walking *vs* Sitting | 15 seconds | 30 seconds | 60 seconds | 90 seconds | 120 seconds |
|---|---|---|---|---|---|
| *Look* | t=2.2, p>0.05 | t=2.7, p>0.05 | t=3.2, p>0.05 | t=1.1, p>0.05 | t=0.5, p>0.05 |
| *Read* | t=0.9, p>0.05 | t=-0.7, p>0.05 | t=-0.8, p>0.05 | t=0.03, p>0.05 | t=-0.2, p>0.05 |
| *Solve simple* | t=-0.8, p>0.05 | t=-3.7, p>0.05 | t=-7.1, p>0.05 | t=-8.4, p<0.05 | t=-10.8, p<0.01 |
| *Solve hard* | t=-1.5, p>0.05 | t=-2.2, p>0.05 | t=-6.9, p>0.05 | t=-11.7, p<0.001 | t=-14.6, p<0.001 |
